# Cost analysis of machine and manual reprocessing of transvaginal ultrasound probes

**DOI:** 10.3205/dgkh000511

**Published:** 2024-11-05

**Authors:** Denise Kiefner, Hicham Benkhai, Sandra Lemanski, Marc Thanheiser, Axel Kramer

**Affiliations:** 1 Institute of Hygiene and Environmental Medicine, University Medicine Greifswald, Greifswald, Germany; 2 Department of Psychology, Chair of Health and Prevention, University of Greifswald, Greifswald Germany; 3 Division Hospital Hygiene, Infection Prevention and Control, Department of Infectious Diseases, Robert Koch Institute, Berlin, Germany

**Keywords:** TVUS reprocessing, manual reprocessing, machine reprocessing, economic analysis, process time, personnel costs, material costs, survey results on reprocessing practices

## Abstract

**Objective:**

This study aims to provide additional support for the equipment needed in hospitals and medical practices for reprocessing transvaginal ultrasound probes (TVUS) through an economic analysis comparing manual and automated reprocessing methods. A questionnaire survey was also conducted in hospitals and medical practices to analyze the current practice of TVUS reprocessing.

**Methods:**

The economic analysis compared four manual reprocessing methods using disinfection wipes and one automated device-based disinfection method using hydrogen peroxide vapor. The working times were measured with a stopwatch and complemented by passive working time (disinfection exposure time or machine cycle duration). The personnel costs for the working time were calculated and combined with the calculated acquisition and material costs to determine the total process costs.

**Results:**

The economic analysis revealed that machine disinfection is not only time-saving but also more cost-effective per reprocessing cycle compared to two manual methods using wipes for cleaning and disinfection, where the disinfectant is applied to the wipe before use. However, two methods using ready-to-use (rtu) disinfection wipes from a container were more cost-effective. It is important to note that all wipe disinfection methods would incur additional costs due to the lack of validation. The additional costs for validation could not be calculated due to a lack of experience, making a final cost assessment for wipe disinfection methods currently impossible.

Despite extensive efforts to send the survey to hospitals and medical practices through three professional societies and attempts to acquire participants via a publication, only 35 institutions participated. Except for one case, all reprocessed manually. The survey revealed a deficit in knowledge regarding TVUS reprocessing. Manual reprocessing had not been validated despite national legal requirements existing since 2002.

**Conclusion:**

As long as manual reprocessing is not validated in all steps, only machine reprocessing is ethically acceptable for patient safety. Even if manual wipe disinfection is validated, machine reprocessing offers higher patient safety, since deviations from the validated SOP cannot be excluded during manual execution. Machine reprocessing should always be preferred for occupational safety reasons.

Since the process costs for methods involving the application of the disinfectant to the wipe before disinfection were higher than for the machine method, the latter is preferred in this comparison. It is not possible to determine whether the overall process is economically superior to machine reprocessing because the cost calculation for rtu disinfection wipes does not include the entire process of reprocessing, including the legally required validation. Due to the better standardization of the machine reprocessing process, along with increased procedural, worker, and patient safety, machine reprocessing should be preferred regardless of process costs, especially if the economic difference is not significant. Additionally, avoiding disposable wipes is beneficial in terms of sustainability.

The lack of knowledge regarding national legal requirements and recommendations for TVUS reprocessing is a reason why the principles of reprocessing were not adhered to in several practices. Therefore, it is necessary to convey the basic knowledge of reprocessing during medical studies, followed by further training during specialization. Persons tasked with reprocessing must have the required education or participate in specialized training to demonstrate current knowledge (§ 8 Sect. 7 Medical Device Regulation).

## Zusammenfassung

**Zielsetzung:**

Mit Hilfe einer Wirtschaftlichkeitsanalyse von manueller und apparativer Aufbereitung transvaginaler Ultraschallsonden (TVUS) sollte eine zusätzliche Hilfestellung für die zur Aufbereitung von TVUS erforderliche Ausrüstung von Krankenhäusern und Arztpraxen gegeben werden. Begleitend sollte eine Fragebogenerhebung in Krankenhäusern und Arztpraxen durchgeführt werden, um die derzeitige Praxis der Aufbereitung von TVUS zu analysieren.

**Methode:**

Die Wirtschaftlichkeitsanalyse wurde für vier Verfahren der manuellen Aufbereitung mit Desinfektionstüchern (Wipes) und ein automatisiertes apparatives Desinfektionsverfahren auf Basis von Wasserstoffperoxidverneblung durchgeführt. Die Arbeitszeit wurden mittels Stoppuhr bestimmt und durch die passive Arbeitszeit (Einwirkungszeit der Desinfektion bzw. Zeitdauer des Programmdurchlaufs der Maschine) komplettiert. Für die aufgewendete Arbeitszeit wurden die Personalkosten kalkuliert und durch Addition der kalkulierten Anschaffungs- und Materialkosten der Gesamtprozess berechnet.

**Ergebnisse:**

Die Wirtschaftlichkeitsanalyse ergab, dass die maschinelle Desinfektion nicht nur zeitsparender als die manuelle Wischdesinfektion ist, sondern pro Aufbereitung im Vergleich zu zwei manuellen Verfahren mit Einsatz von Tüchern zur Reinigung und zur Desinfektion, bei denen das Desinfektionsmittel vor der Anwendung auf das Tuch gegeben wird, auch kostengünstiger ist. Dagegen erwiesen sich zwei Verfahren mit zur fortlaufenden Entnahme aus einem Behältnis befindliche ready-to-use (rtu)-Desinfektionswipes als kostengünstiger. Hierbei ist zu beachten, dass für alle Desinfektionsverfahren mittels Wipes aufgrund der jeweils noch ausstehenden Validierung zusätzliche Kosten berücksichtigt werden müssten. Die hierfür entstehenden zusätzlichen Kosten konnten mangels Erfahrungswerten nicht einberechnet werden; somit ist derzeit für Desinfektionsverfahren mittels Wipes keine abschließende Kostenabschätzung möglich.

Trotz umfangreicher Bemühungen durch Versendung der Umfrage an Kliniken und Arztpraxen, an drei Fachgesellschaften und den Versuch, durch eine Veröffentlichung Teilnehmer zu akquirieren, nahmen nur 35 Einrichtungen an der Umfrage teil. Mit einer Ausnahme wurde nur manuell aufbereitet. Die Befragung ergab ein Defizit im Kenntnisstand zur Aufbereitung von TVUS. In keinem Fall war die manuelle Aufbereitung, trotz der seit 2002 existierenden nationalen rechtlichen Vorgaben in der MPBetreibV, bisher validiert worden.

**Schlussfolgerung:**

Solange die manuelle Aufbereitung nicht in allen Teilschritten validiert ist, ist im Interesse der Patientensicherheit nur die maschinelle Aufbereitung ethisch vertretbar. Selbst wenn die manuelle Wischdesinfektion validiert worden ist, bietet die apparative Aufbereitung die höhere Patientensicherheit, weil bei manueller Durchführung Abweichungen von der auf der Grundlage der Validierung definierten SOP nicht auszuschließen sind. Auch aus Arbeitsschutzgründen ist der apparativen Aufbereitung immer der Vorzug zu geben.

Da die Prozesskosten bei beiden Verfahren mit Auftragung der Desinfektionslösung auf das Wipe vor der Desinfektion höher waren als bei dem apparativen Verfahren, ist letzteres in diesem Vergleich zu bevorzugen. Da bei den rtu- Desinfektionswipes die Kostenkalkulation nicht den Gesamtprozess der Aufbereitung einschließlich der gesetzlich geforderten Validierung abbildet, lässt sich nicht ableiten, ob der Gesamtprozess ökonomisch der apparativen Aufbereitung überlegen ist. Da die Durchführung der apparativen Aufbereitung besser standardisierbar ist, verbunden mit erhöhter Verfahrens-, Arbeitnehmer- und Patientensicherheit, ist unabhängig von den Prozesskosten der apparativen Aufbereitung der Vorzug zu geben, zumal wenn sich die Wirtschaftlichkeit nicht erheblich unterscheidet. Auch im Hinblick auf Nachhaltigkeitsaspekten ist eine Vermeidung von zu entsorgenden Wipes zu begrüßen.

Die z.T. fehlende Kenntnis der nationalen gesetzlichen Vorgaben und Empfehlungen zur Aufbereitung von TVUS ist als ein Grund dafür anzusehen, dass die Grundsätze der Aufbereitung in mehreren Praxen nicht eingehalten wurden. Daraus ergibt sich die Notwendigkeit der Vermittlung des Basiswissens zur Aufbereitung bereits im Medizinstudium mit nachfolgender Vertiefung in der Weiterbildung. Auch die mit der Aufbereitung beauftragten Personen müssen über eine erforderliche Ausbildung verfügen bzw. es kann für den Nachweis der aktuellen Kenntnis die Teilnahme an fachspezifischen Fortbildungsmaßnahmen berücksichtigt werden (s. § 8 Abs.7 MPBetreibV).

## Introduction

The reprocessing of medical devices (MDs) intended for almost sterile or sterile use must be performed with validated procedures to ensure patient safety [1], [2]. This includes all steps of reprocessing, such as pre-cleaning, cleaning, disinfection, removal of cleaning and disinfectant residues, and final sterilization for sterile MDs. The type testing for machine cleaning-disinfection procedures is regulated by standards. Automated reprocessing methods are validated according to similar principles. Manual reprocessing with a wipe procedure must also validate all steps such as precleaning, cleaning, intermediate rinsing, disinfection, and final rinsing [3], [4]. The procedures must be documented in a standard operating procedure (SOP), and the personnel must be trained accordingly. Although there is a guideline for manual reprocessing [5], MDs requiring wipe disinfection are excluded from its scope. However, manual immersion disinfection is included and considered validatable, because the experimental process of immersion is covered by the test methods of the Association for Applied Hygiene (VAH), ensuring reproducible application by the operator on-site [1], [6], [7]. It is crucial to use instrument disinfectants with proven effectiveness, such as those listed by the VAH [8]. If a validated machine-reprocessing method is available, manual reprocessing of semi-critical and critical MDs requires proof of equivalent performance to the machine method [1].

In Germany, TVUS are currently reprocessed either manually with wipes or using automated methods, both required as high-level disinfection [1], [9]. An economic analysis of both methods was necessary to verify the common perception that manual reprocessing is more cost-effective. Since no manual wipe method has been validated despite national legal requirements since 2002, only the costs of the reprocessing process, including material costs, could be determined for manual reprocessing. In contrast, the costs for type testing of the machine method are included in the purchase price. For the selected device for the economic analysis, the manufacturer states that no validation after installation is required.

A survey was conducted to determine the current reprocessing practice for TVUS.

## Method

### Survey

The survey was conducted as a paper-pencil questionnaire sent to gynecology and obstetrics practices (randomly selected) and as an online version, anonymously and voluntarily, with written consent. The survey could be completed in about 5 minutes. Participants were informed about the purpose, the fact that it was voluntary, the opportunity to ask questions, and data handling. The online survey was implemented using the “Unipark” survey tool (Questback GmbH, https://www.unipark.com) and shared with the German Society for Gynecology and Obstetrics e.V., the German In-Vitro-Fertilization Register, and the German Society for Ultrasound in Medicine. Due to the low response rate, an interview contribution was initiated in the journal “Hospital and Management” to motivate further participation in the online survey. The questionnaire (see Attachment 1) was tested for comprehensibility with three female and three male experts in the field of gynecology and obstetrics and adjusted after validation.

In addition to asking whether TVUS was reprocessed manually or using machines, information on process design, including known recommendation bases, and the implementation of hygiene in the work environment was requested.

### Economic analysis

#### Examined reprocessing methods

Two ready-to-use (rtu) disinfection wipes (methods A and B), a wipe on which the disinfection solution is applied immediately before use (method C), a 3-wipe system for cleaning, disinfection, and final re-cleaning (method D), and an automated chemothermal disinfection method using hydrogen peroxide as a biocide (method E) were analyzed (Table 1 (Tab. 1)). The latter includes manual cleaning, chemothermal disinfection, machine drying, and digital documentation of critical process parameters. Methods A–D are recommended by the manufacturer for reprocessing various ultrasound probes, while method E is recommended only for TVUS. All methods were used according to the manufacturer’s instructions.

The economic analysis simulated manual and automated reprocessing in a room of the Department of Obstetrics and Gynecology at the University Medicine Greifswald, where the automated reprocessing method was installed.

In methods A (Cleanisept^®^ Wipes forte; Dr. Schumacher GmbH, Malsfeld, Germany) and B (Incidin^®^OxyWipe S Desinfektionstücher; Ecolab Deutschland GmbH, Wallisellen; Germany), a wipe soaked with disinfectant from a bulk pack is used. In method C (Tristel Duo^®^; Tristel GmbH Berlin, Germany), the wipe is soaked with the disinfectant immediately before use. Method D (Tristel Trio^®^ Wipes System; Tristel GmbH, Berlin, Germany) is a 3-wipe system consisting of a separate wipe for cleaning, disinfection, and final cleaning/drying. Each component allows documentation through a protocol book label with LOT number and expiration date for the three steps until approval by the reprocessing personnel. Documentation is possible, but without critical process parameters. In method E (Trophon2^®^; Nanosonics Ltd., Europe GmbH, Hamburg, Germany), disinfection is performed in a chamber at a minimum of 56°C with 35% hydrogen peroxide vapor (cycle duration including drying: 7 minutes). Pre-cleaning and cleaning are done with a Cleaning Wipe^®^ and validated by checking the residual protein content. After the disinfection cycle, the user is prompted to check if the probe is dry. If not, it must be dried with a Drying Wipe^®^. AcuTrace^®^ enables digital traceability of the probe and user identification using RFID technology. Internal sensors and chemical indicators ensure quality by monitoring process parameters such as time, temperature, and dosage. The user receives the validation report in both digital and analog form, providing process documentation and the basis for subsequent parametric approval.

#### Economic analysis

For manual wipe systems, the same process steps detailed in the recommendation of the Commission for Hospital Hygiene and Infection Prevention at the Robert-Koch Institute Berlin (KRINKO) and the Federal Institute for Drugs and Medical Devices (BfArM) [2] were always simulated to standardize time measurements (Table 2 (Tab. 2)). Intermediate rinsing was omitted, as manufacturers stated that disinfection was adequate without it.

**Figure 1:**
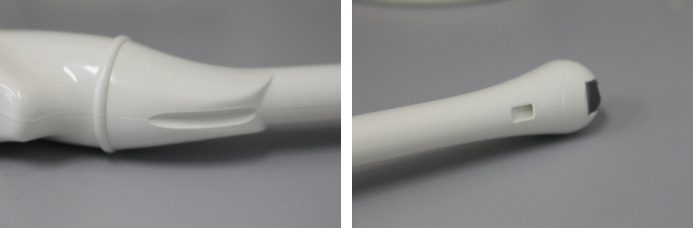
Indentations on the probe

The wiping technique was standardized, starting with pre-cleaning until visible cleanliness of TVUS prepared with ultrasound gel, followed by cleaning and disinfection as per manufacturer’s instructions, until the entire TVUS including the handle are visible wet. Although not specified by the manufacturer, cloths were wrung out or foam was applied to indentations and cleaned with cotton swabs for best cleaning and disinfection results (Figure 1 (Fig. 1)).

The reprocessing procedure for the (semi-)automated method (E) was similarly standardized (Table 3 (Tab. 3)). For the (semi-)automated process, the working path and disposal of consumables were included in the time measurement. Manual cleaning of indentations with cotton swabs was omitted, as sufficient gel residue removal with a cleaning cloth for successful disinfection has been described [10]. No working time was calculated for digital documentation. For printout documentation, the time taken by the printer and the time the responsible person needed to attach the printout to the TVUS cover were measured.

Time measurements were conducted with a calibrated stopwatch. Each reprocessing step was performed ten times to calculate the average working time. Active and passive times were distinguished, i.e., exposure time of disinfectants and glove disposal time or machine cycle time. The probe was always covered with a protective sheath and prepared with ultrasound gel to simulate a practical scenario.

Personnel costs for the time spent reprocessing were calculated for nurses (pay grade KR 7, level 3), doctors (pay grade Ä2, specialist), and medical assistants (MFA) based on the collective bargaining agreement for public service in the federal states (TV-L) and the salary tariff agreement for MFA/nurse assistants (§ 3). No above-tariff wage agreements were considered. Personnel costs per reprocessing were calculated by multiplying the working time with the personnel costs.

Material costs were determined based on specialist trade prices and manufacturer list prices (as of February 2021). Prices are dynamic and vary significantly depending on whether materials are procured from individual traders or through the purchasing department of a university clinic. Prices for consumables, disinfectants, operating costs, and device prices are net prices from the specified suppliers or manufacturers.

One-time acquisition costs such as device or operating costs were allocated over a usage period of five or ten years. This also applies to the costs of validations and maintenance of the automated reprocessing system. A usage period of one year was set for the protective glasses needed for manual reprocessing.

Energy costs were calculated with a net price of €0.23 per kilowatt-hour (kWh).

Process costs for the automated reprocessing method were calculated assuming ten reprocessing cycles per day on 256 working days, resulting in 2,560 applications per year.

Finally, personnel, material, and total costs per reprocessing were determined.

## Results

### Survey

There was no response to the version distributed as online link in the “Unipark” survey tool or to direct inquiries to gynecological societies. Following postal distribution of the paper-pencil version, 19 participants responded. 16 facilities took part in the online survey in the magazine Krankenhaus und Management. Thus, 35 questionnaires from 25 hospitals and 10 medical practices included in the evaluation.

Thirty-three (33) participants used disinfection wipe procedures for TVUS reprocessing. Immersion disinfection and an automated method were each used once. The main reason for choosing manual procedures was the short process duration (n=28), followed by the probe manufacturer's recommendation (n=15) and lower costs (n=7). Documentation and legal security were cited as reasons for automated reprocessing.

Manual reprocessing revealed the following deficiencies: No designated person for reprocessing approval (n=23), no batch number assignment (n=22), no documentation of process parameter measurements with at least 5-year retention (n=21), no cleaning after wipe disinfection (n=16), no approval decision (n=13), no additional contamination removal, no visual inspection after reprocessing, and no use of VAH-listed wipes for disinfection (n=3 each), and no removal of gel residues with a dry cloth after removing the protective cover (n=2). 

Deficiencies in knowledge of the required effect spectrum were indicated: bactericidal (n=32), fungicidal (n=25), levurocidal (n=23), mycobactericidal (n=17), and tuberculocidal (n=12). Instead of virucidal (e.g., effective against Papovaviruses), four times limited virucidal and six times limited virucidal Plus were indicated as required. Although sporicidal effectiveness is not required, it was indicated 21 times as required.

In 26 institutions, no residual protein determination was performed after reprocessing. Six institutions used a commercial swab test, three sent the test to a laboratory. Testing intervals were monthly (n=4), quarterly (n=1), semi-annually (n=3), or annually (n=1).

The ultrasound (US) probe was reprocessed after each use. However, hand contact surfaces such as the keyboard and handle were only disinfected daily after the last examination in 13 practices. The US gel bottle was disinfected after each use in ten practices, in the remaining practices only daily after the last use. Only five respondents disinfected the US device, including cable and plug, after each use. In 23 practices, this was done daily after the last examination, and weekly in seven practices. In 18 institutions, no disinfection of the US device was performed before the first use of the day, 13 practices disinfected the entire US device including cursor and keyboard, and in four practices, only the probe was disinfected. The majority (n=23) reported daily disinfection of all work surfaces.

Hand antisepsis was reported as follows: before each examination (n=30), after each examination (n=27), upon entering the examination room (n=12), and at the end of the work shift (n=1).

In 28 institutions, the seat was covered with paper, which was changed after each patient in 27 institutions and daily in one institution. In five institutions, a wipe disinfection was performed instead of a cover after each patient. In two institutions, a textile cover was used, changed daily.

The joint recommendation of the KRINKO/BfArM on the hygiene requirements for reprocessing medical devices [2], which is the basis for every reprocessing, was only known to 18 institutions. Thirteen (13) institutions followed the hygiene recommendations in sonography and endosonography of the German Society for Ultrasound in Medicine [11].

#### Economic analysis

**Figure 2:**
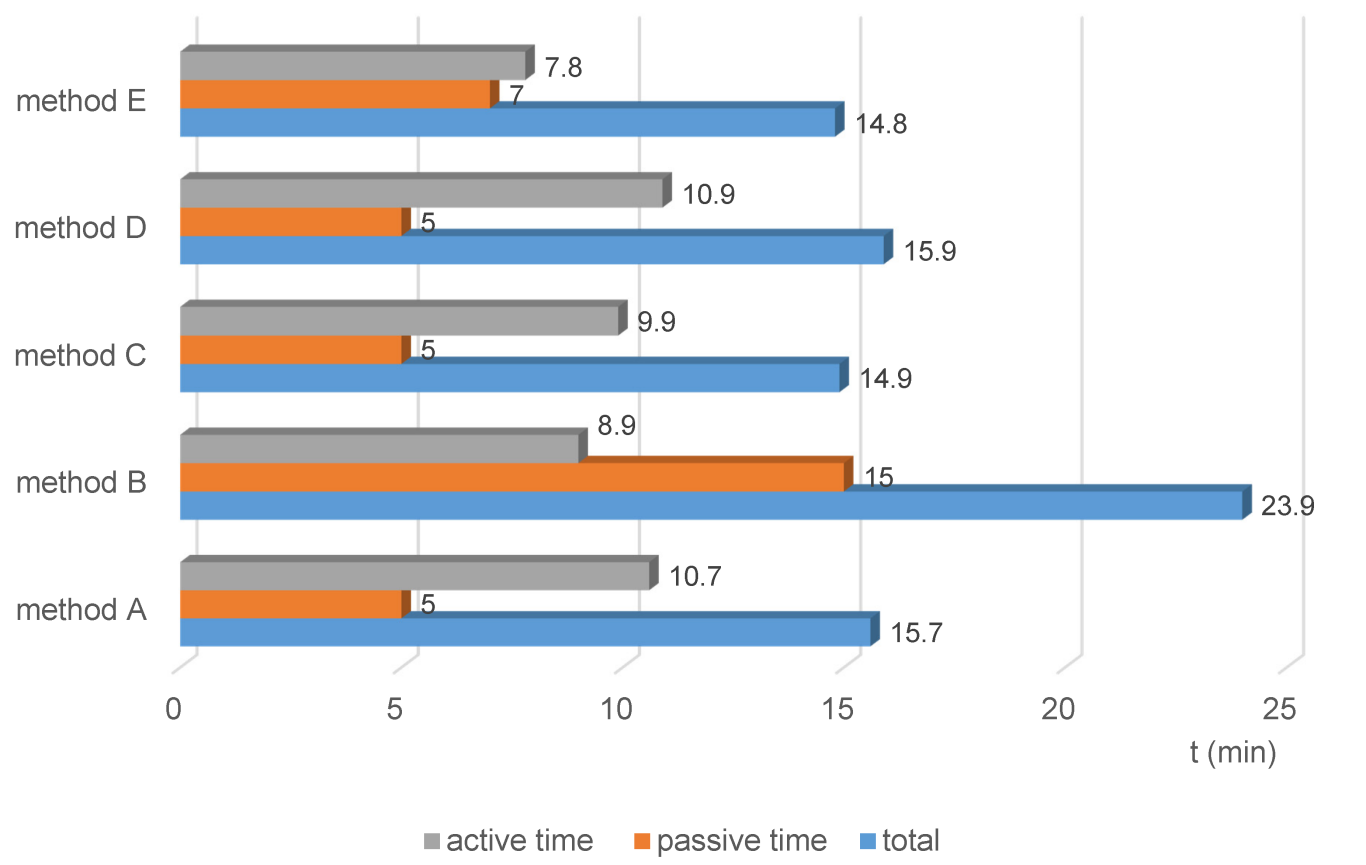
Figure 2 : Total process time for reprocessing

In total active and passive time, the automated method E was the shortest (14.8 min), method B the longest (23.9 min) (Figure 2 (Fig. 2)).

**Figure 3:**
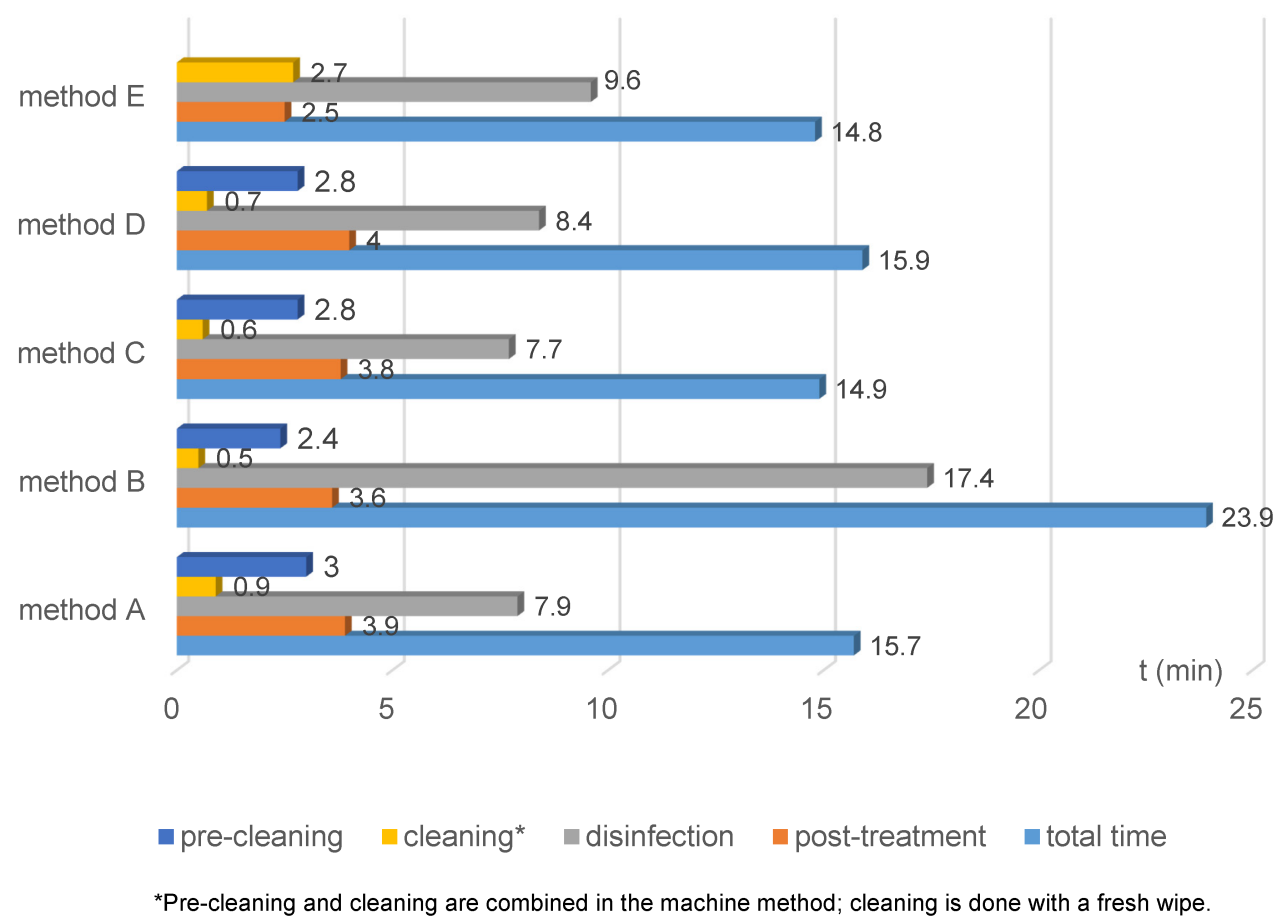
Time duration of reprocessing steps

In Figure 3 (Fig. 3), the processing time is broken down into pre-cleaning, cleaning, disinfection, and post-treatment. In the automated method, the disinfection duration was longest except for method B; the shorter times for cleaning and post-treatment led to the overall shorter time. Pre-cleaning and cleaning are combined in the machine method (see p. 19 and p. 24 [12]). The Cleaning Wipe^®^ replaces pre-cleaning, and a manual disinfection wipe was used before chamber disinfection. Thus, additional wipe disinfection was performed before actual chamber disinfection.

**Figure 4:**
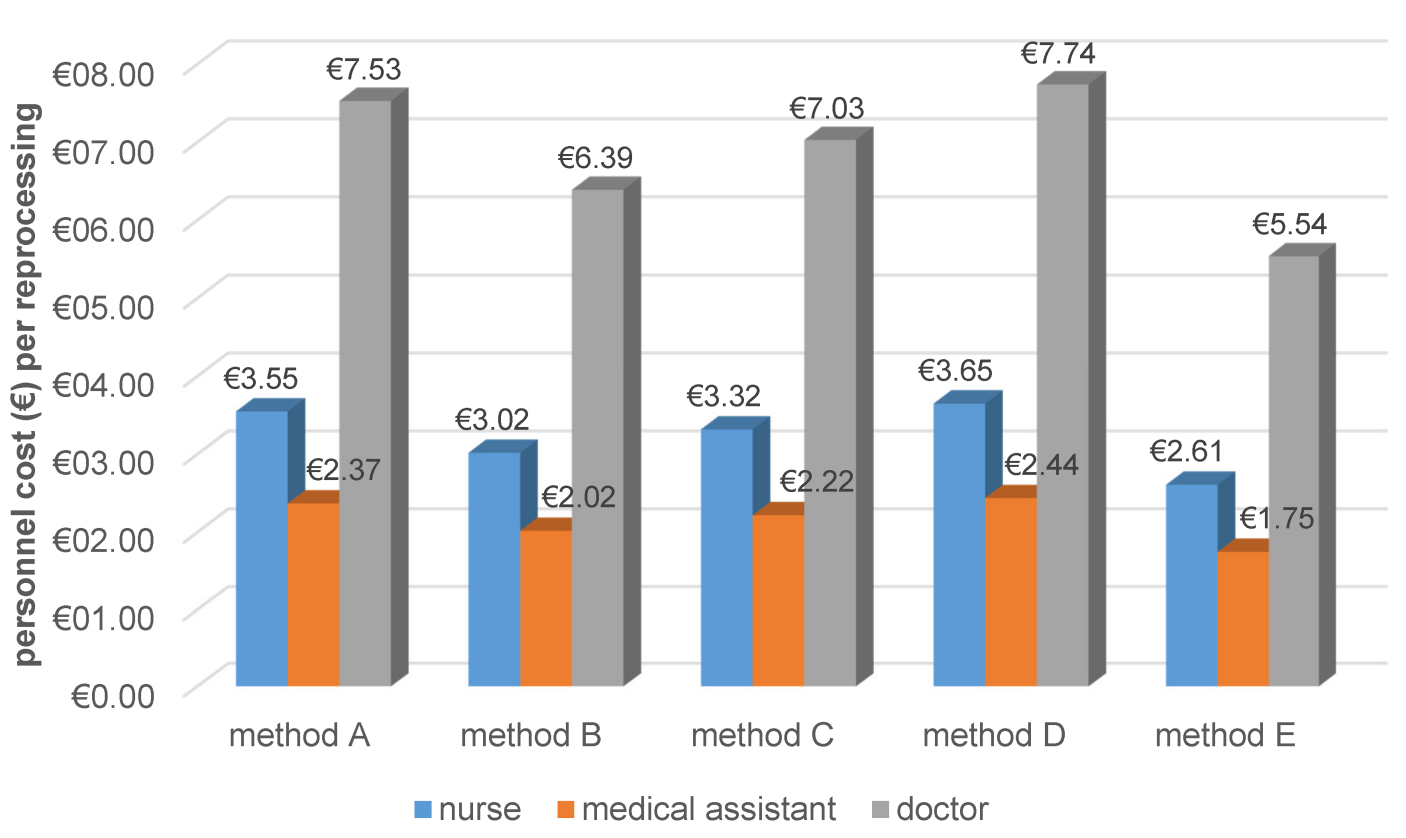
Figure 4 : Personnel costs per reprocessing

Personnel costs per reprocessing depend on the professional group performing the reprocessing. Regardless of the professional group, personnel costs were lowest for the automated method (Figure 4 (Fig. 4)). 

Material costs per reprocessing were highest for method D. For the automated method E, costs depend on whether a 5 or 10-year usage period is assumed (Table 4 (Tab. 4)). 

**Figure 5:**
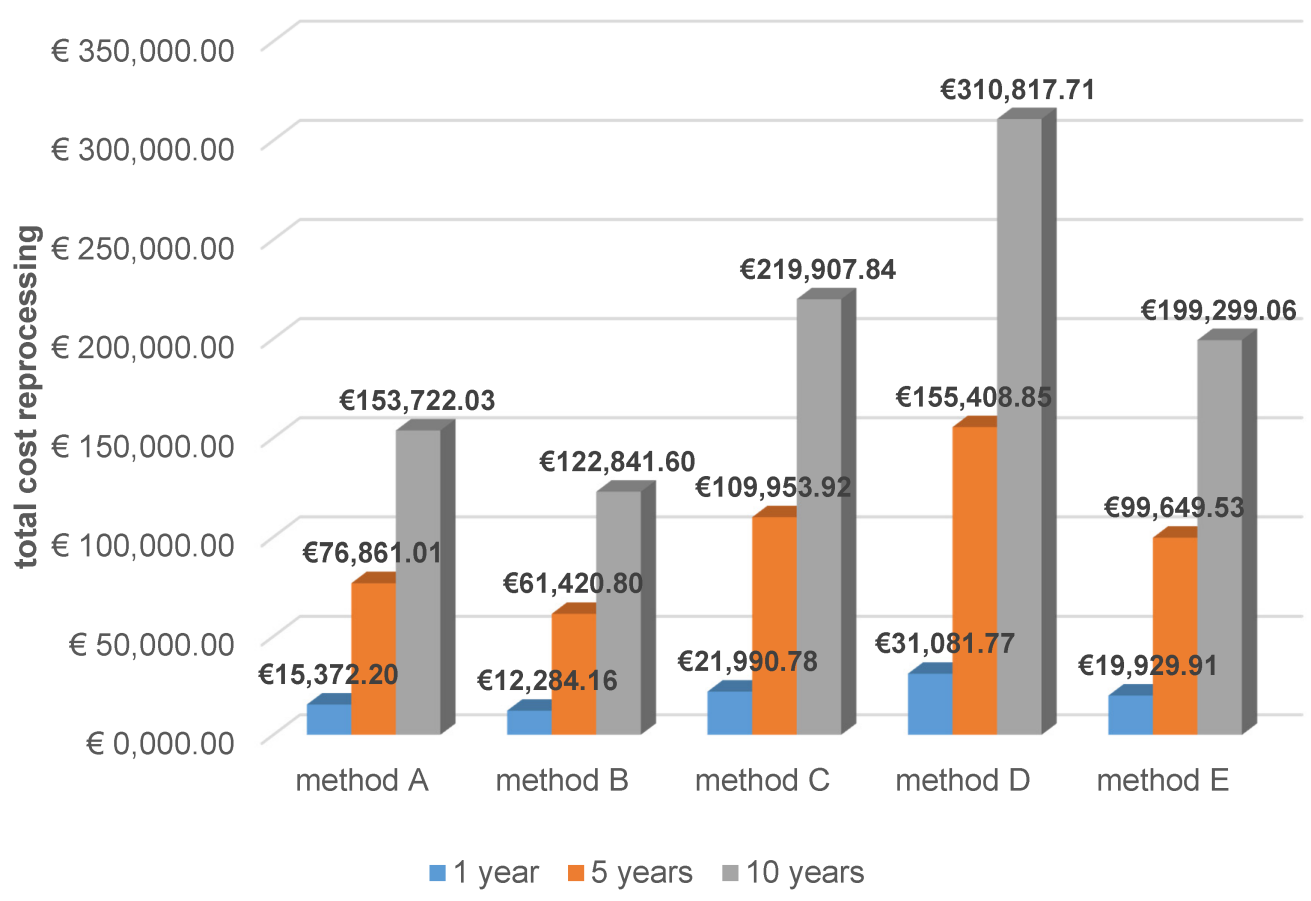
Total reprocessing costs cumulated for one, five, and ten years (for method E, device costs are amortized over 10 years)

Concerning total personnel and material costs, methods A and B were significantly less expensive than methods C, D, and E, regardless of whether costs were cumulated over one, five, or ten years (Figure 5 (Fig. 5)).

Due to the extensive measurements and calculations (30 pages), they are available upon request or in the appendix of the dissertation upon which this paper is based [12]. 

## Discussion

### Method

This survey aimed to provide an overview of the current reprocessing situation in Germany. It was important not to suggest answers to the respondents, which is why specific actions during disinfection were queried rather than process evaluations.

The questionnaire revealed several methodological weaknesses in hindsight. Strict anonymity prevented questions about the professional field. Thus, it remained unclear whether a specialist doctor or a medical assistant/nurse filled out the survey. The paper-pencil format was less appealing than the online tool, possibly contributing to the initial low response rate. Some questionnaires contained multiple answers due to ambiguous questioning. Additionally, it would have been insightful to know how respondents informed themselves about the current reprocessing situation and which recommendations they followed.

The economic analysis simulated reprocessing in a room of the Department of Obstetrics and Gynecology at the University Medicine Greifswald, where the automated reprocessing method was installed. The standardization of measurements by taking the same paths and using a calibrated clock makes the time measurements realistic, but they are not directly transferable to everyday practice. To achieve complete wetting of the probe with wipes, indentations and grooves were wetted separately with a cotton swab. The time for this is not typically expended in practice.

The omission of manual cleaning of indentations and grooves before automated disinfection with hydrogen peroxide is critical, as residues such as protein, blood catalase, and salts can make disinfection success dubious [13]. This time effort should have been included in the economic analysis.

The different yield of various wipe products concerning the amount of disinfectant released also impacted the process duration, requiring a varying number of wipes to achieve complete wetting of the probe.

Personnel costs were calculated based on three years of professional experience, which allows only an indicative calculation. Material costs could only be estimated, as specialist trade and manufacturer list prices vary and change dynamically.

Although the calculated process costs provide only an orientation, the costs of the reprocessing methods can be compared due to the similar methodological approach.

In the cost calculation of manual methods, the effort for initial validation could not be considered due to the lack of experience. This also applies to the follow-up costs associated with validation documentation and periodic residual protein determination.

### Results

#### Survey

The low participation in the survey likely reflects the time overload in daily practice, possibly overlapped by low interest in the topic. Knowledge deficits about TVUS reprocessing may also play a role, as evidenced by nine incomplete questionnaires.

Unfortunately, the sample size of 35 allows only an indicative assessment. Additionally, 25 of the participants were from hospitals, making the survey non-representative for smaller practices.

The survey revealed that almost exclusively wipe procedures were used, which have not been validated, contrary to the Medical Devices Operator Ordinance [1], and partly did not cover the recommended effect spectrum [1]. The main reason for choosing wipe disinfection was the short process duration. However, the economic analysis showed the shortest process duration for automated reprocessing. Costs were also cited as a reason for choosing manual methods. While this is true for acquisition costs, the comparison of cumulative costs for one, five, and ten years showed that automated reprocessing was cheaper than using two of the wipe systems. Two other wipe systems were less expensive than the automated method, but it remains unclear what additional costs arise from the required validation and the time effort when the validated reprocessing process is routinely performed based on an SOP.

#### Economic Analysis

The automated reprocessing method had the shortest process duration overall. Differences in the generally similar working times for manual reprocessing resulted from the different handling or yield of disinfectant solution from the wipes and different exposure times. Since no drastic cost differences were found between the automated and two manual reprocessing methods with specified steps, and even two more wipe methods were less expensive but susceptible to additional costs from validation, a general preference for automated reprocessing can be concluded due to higher procedural safety from better standardization. After using a ready-to-use wipe with microbicidal, levurocidal, sporicidal, and virucidal effectiveness, representatives of vaginal, pharyngeal, and skin flora were detected on TVUS in 10.6% of samples [14]. Using the same 3-wipe system in a cardiology unit caused two outbreaks by the same clone and a third outbreak by a new clone of an extended-spectrum beta-lactamase-producing Enterobacter cloacae after transesophageal echocardiography [15]. After switching to automated reprocessing, the outbreak ended.

Validated reprocessing guidelines and quality-assured implementation are crucial, as risk assessments show. A computer simulation calculated the infection risk from contaminated TVUS at 1–6% depending on the pathogen [16]. The Scottish National Health Services analyzed the infection risk after endocavitary ultrasound examinations from 2010–2016. The post-interventional infection risk from TVUS was significantly higher (factor 141) for positive microbiological findings and antibiotic prescriptions compared to a reference population of other hospitalized gynecology patients. During this period, there was no standardized work instruction for reprocessing ultrasound probes in Scotland, leading the authors to emphasize the need for standardized quality-controlled reprocessing guidelines [17]. Due to higher procedural safety, the Charité University Medicine Berlin received second place for the German Patient Safety Award 2020 for introducing the selected automated method for economic analysis [18].

The knowledge deficits from the survey on quality-assured reprocessing of TVUS highlight the need to convey basic reprocessing knowledge during medical school. The importance of consolidating this knowledge during further training, such as in curricula for hygiene officers, should be emphasized. Critically, the indicated deficiencies in disinfecting the ultrasound device, including the gel bottle, cable, and plug, are concerning, since *Staphylococcus aureus* and *Pseudomonas putida* were detected on TVUS keyboards, and *Acinetobacter *spp., *Enterococcus (E.) faecium*, and* E. faecalis* were found on ultrasound devices [19]. An environmental study showed that not only the ultrasound probe was contaminated with blood and bacteria (57% and 46%) but also the ultrasound housing (75% and 75%), keyboard (62% and 50%), and most heavily the ultrasound cable (88% and 62%) [20].

Since manufacturer information was sometimes incomplete or incorrect, there is also a need for improvement here.

Besides hygiene safety and, subsequently, economic efficiency, resource consumption must be minimized in any disinfection method. The automated method likely has lower resource consumption for the following reasons: No waste of plastic packaging for wipes, no used disinfectant wipes waste, no environmental impact from the immediate complete breakdown of hydrogen peroxide into water and oxygen after disinfection, lower resource usage in producing hydrogen peroxide compared to quaternary ammonium compounds combined with NaOH, inorganic peroxides, peracetic acid, and chlorine dioxide, and no additional cleaning wipe needed to remove disinfectant residues after wipe disinfection.

## Conclusions

The lack of knowledge of national legal requirements and recommendations for reprocessing MDs is a reason why reprocessing principles were often not followed. Since the practice owner or the medical director in hospitals is responsible for reprocessing MDs, ensuring validated reprocessing of MDs is imperative to ensure patient safety.

## Limitations

Before simulating automated disinfection, manual cleaning of the probe’s indentation was omitted as it was not required by the manufacturer’s instructions. Since the effectiveness of hydrogen peroxide is significantly affected by residues such as protein, salts, and ultrasound gel, this cleaning step is considered essential in practice [13]. The time required for this is 9.2 s and does not significantly affect the economic analysis.

## Notes

### Competing interests

The authors declare that they have no competing interests.

### Author’s ORCID

Axel Kramer: 0000-0003-4193-2149

## Supplementary Material

Questionnaire

## Figures and Tables

**Table 1:**
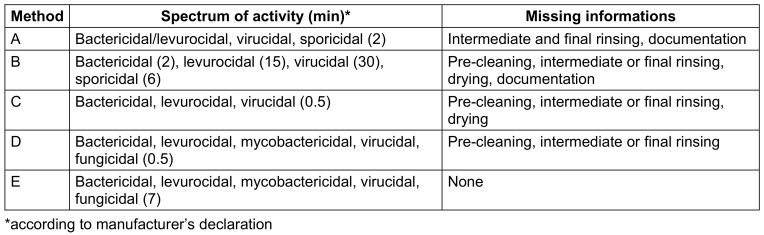
Characteristics of methods selected for economic analysis

**Table 2:**
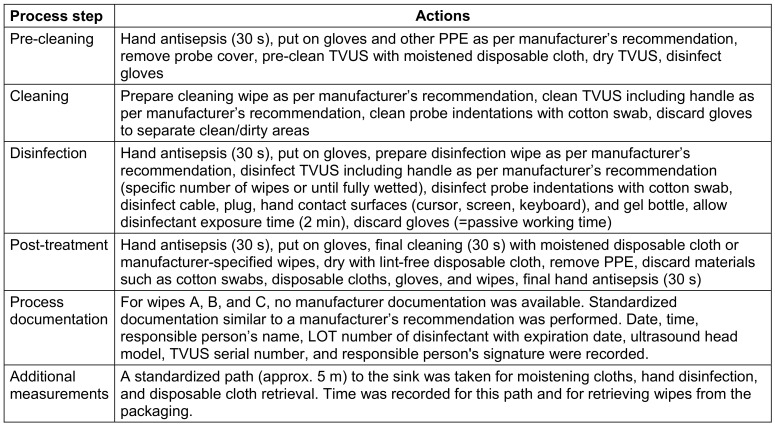
Standardized procedure for manual reprocessing (methods A–D)

**Table 3:**
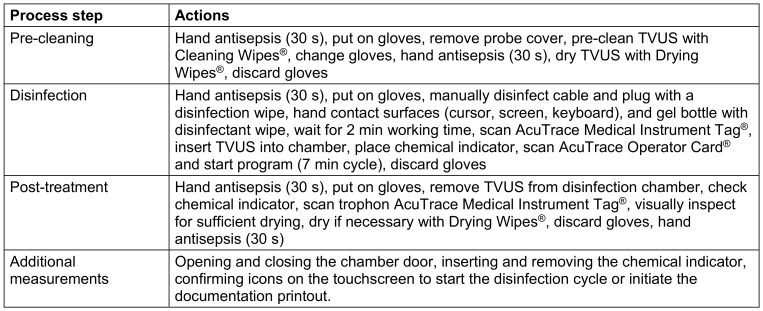
Standardized procedure for automated reprocessing (method E)

**Table 4:**
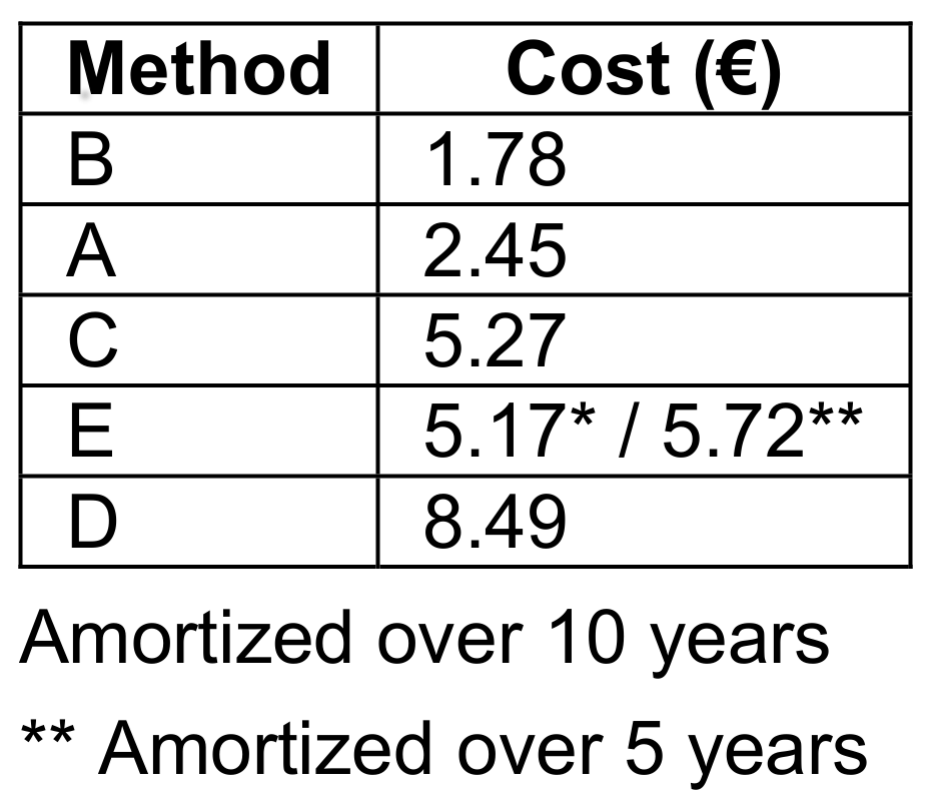
Material costs per reprocessing
