# Supplementary material for: Cost analysis of machine and manual reprocessing of transvaginal ultrasound probes
Source: GMS Hyg Infect Control. 2024 Nov 5;19:Doc56. doi: 10.3205/dgkh000511 (PMC11626639; doi:10.3205/dgkh000511)
Supplement: Questionnaire [file HIC-19-56-s-001.pdf]

Supplement
Questionnaire

Request for participation in a survey on reprocessing transvaginal ultrasound probes

Dear participants,
This survey is part of my doctoral project in which I am experimentally modeling an economic comparison between manual reprocessing of transvaginal ultrasound probes (various methods) and automated reprocessing.

The aim of this survey is to record the current situation regarding the reprocessing of transvaginal ultrasound probes.

The survey is anonymous, voluntary and takes about 5 minutes. To ensure anonymity, we ask that you do not enter any names or other personal data on the questionnaire, as otherwise it cannot be evaluated.
If you decide to take part, you are free to cancel the survey at any time without giving reasons. All data collected will be treated confidentially and will only be accessible to project staff. When results are made public in reports, articles and presentations, the anonymization and summarization of all data means that no conclusions can be drawn about your person or institution.

If you have any questions, please contact Prof. Dr. med. Axel Kramer, responsible supervisor of the dissertation, Institute of Hygiene and Environmental Medicine at the University Medical Center Greifswald (e-mail: axel.kramer@med.uni-greifswald.de; telephone: 03834-864820).

Thank you very much for your support

Denise Kiefner, student at the Faculty of Medicine of the University of Greifswald

Questionnaire

If several answers are possible, this is noted.

1. Do you work in a private gynecological practice or in a hospital?

□ Hospital
□ Practice
□ Fertility center

2. Which method of disinfection do you use?

□ Manual wiping procedure using a cloth
□ Manual immersion method
□ Mechanical procedure

3. Why do you disinfect your probe manually? (multiple answers possible)

□ Short process duration
□ Approval by local authorities
□ Lack of space
□ Spectrum of effectiveness
□ Recommendation of the probe manufacturer
□ Costs
□ other:________________________

4. For what reasons do you disinfect your probe mechanically? (multiple answers possible)

□ Short process duration
□ Spectrum of efficacy
□ Automation and reproducibility
□ Validability of the process
□ Documentation and legal certainty
□ Recommendation of the probe manufacturer
□ Occupational health and safety
□ other:_________________________

5. Which product/device do you use?

□ Cleanisept® Wipes forte (Dr. Schumacher)
□ Incidin® OxyWipes S (Ecolab)
□ Tristel Duo® ULT+Tristel Dry Wipes®
□ Tristel Trio® Wipes System
□ trophon® (Nanosonics)
□ No ready-made wipes, solution is prepared by the user and applied with a wipe
□ other:__________________________

6. Which of the above steps do you omit when reprocessing the probe?
(multiple answers possible)

□ Removing the protective cover and most of the gel residue with a dry cloth
□ Remove any visibly remaining soiling again with a second cloth
□ In the case of manual disinfection, follow the instructions for use, in particular concentration and exposure time from the VAH list
□ Remove disinfectant residues with water of at least drinking water quality
□ Prevention of recontamination
□ Visual inspection
□ Release decision
□ Designated person for release
□ Allocation of batch number
□ Documentation of the measured values of the process parameters and storage for at least 5 years

7. How would you classify the transvaginal ultrasound probe and how would you prefer to reprocess it?

□ semi-critical A- manual reprocessing
□ semi-critical A- automated reprocessing
□ semi-critical B- manual reprocessing
□ semi-critical B- mechanical reprocessing

8. What spectrum of efficacy of the disinfectant for transvaginal US probes do you consider necessary? (multiple answers possible)

□ bactericidal
□ tuberculocidal
□ mycobactericidal
□ virucidal
□ limited virucidal plus
□ limited virucidal
□ fungicidal
□ Levurocidal (e.g. *Candida albicans*)
□ sporicidal

9. What exposure time do you adhere to?

□ Specification of the manufacturer of the disinfection process
□ Specification of the manufacturer of the probe
□ According to the VAH list

10. Has your reprocessing procedure been validated in accordance with MPBetreibV § 8 Para.1 by qualified specialists at the operator's site?

□ yes
□ no
□ is unknown to me

11. Do you use a test to determine residual protein?

□ yes, a commercial swab test
□ send to a laboratory for determination
□ no

12. If you carry out the residual protein determination or have it carried out, at what interval?

□ monthly
□ quarterly
□ every six months
□ annually

13. Do you prepare the transvaginal transducer in the same way as the transabdominal transducer?

□ yes
□ no, separate reprocessing instructions

14. Is the US device disinfected before starting work?

□ yes, only the probe
□ yes, the complete device - probe including cursor and keyboard
□ No, no routine disinfection is carried out before starting work

15. Who carries out the basic cleaning of the US device?

□ the user (doctor)
□ the medical assistant(s)
□ the link hygiene nurse

16. How often do you disinfect the probe again in everyday practice?

□ after each use
□ after obvious soiling (e.g., blood)
□ when changing users

17. How do you handle hand contact surfaces, e.g., keyboard and handle?

□ Wipe disinfection every working day after the last examination
□ Wipe disinfection after each examination

18. How do you handle the ultrasound gel bottle?

□ Wipe disinfection every working day after the last examination
□ Wipe disinfection after each examination

19. How do you handle the reprocessing of the US device including cable and plug?

□ Wipe disinfection every working day after the last examination
□ Wipe disinfection weekly
□ Wipe disinfection after each examination

20. If the examination room is cleaned and disinfected, at what intervals is this done?

□ Cleaning of all work surfaces every working day
□ Disinfecting cleaning of all work surfaces every working day
□ Weekly cleaning of all work surfaces
□ Weekly disinfectant cleaning of all work surfaces
□ Cleaning the floor every working day
□ Weekly disinfectant cleaning of the floor
□ No fixed intervals for cleaning/disinfecting the room

21. How do you handle the examination chair?

□ Use of a paper cover with daily change
□ Use of a paper cover with change after each patient
□ Use of a textile cover with daily change
□ Use of a textile cover with weekly change
□ Instead of a protective cover, wipe disinfection of the contact surface after each examination

22. At what intervals do you disinfect your hands? (multiple answers possible)

□ when entering the examination room
□ before each new patient
□ after every examination
□ Only at the end of the work shift

23. Which of the following reprocessing recommendations are you aware of? (multiple answers possible)

□ KRINKO/ BfArM
□ DGKH (German Society for Hospital Hygiene)
□ DGSV (German Society for Sterile Supply)
□ AKI (Instrument Reprocessing Working Group)
□ VAH (Association for Applied Hygiene)
□ DEGUM (German Society for Ultrasound in Medicine)
□ KBV (German Association of Statutory Health Insurance Physicians)
□ RKI (Robert Koch Institute)
□ others: ________________________
